# A Genome Wide Association Study Reveals Markers and Genes Associated with Resistance to *Fusarium verticillioides* Infection of Seedlings in a Maize Diversity Panel

**DOI:** 10.1534/g3.118.200916

**Published:** 2018-12-19

**Authors:** Lorenzo Stagnati, Alessandra Lanubile, Luis F. Samayoa, Mario Bragalanti, Paola Giorni, Matteo Busconi, James B. Holland, Adriano Marocco

**Affiliations:** * Dipartimento di Scienze delle Produzioni Vegetali Sostenibili, Università Cattolica del Sacro Cuore, via Emilia Parmense 84, 29122 Piacenza (Italy); † Department of Crop & Soil Sciences, North Carolina State University, Raleigh, North Carolina 27695; ‡ U.S. Department of Agriculture-Agricultural Research Service, Plant Science Research Unit, Raleigh, North Carolina 27695

**Keywords:** *Fusarium verticillioides*, resistance to seedling disease, Genome Wide Association Studies, Maize, Rolled Towel Assay, SNPs

## Abstract

*Fusarium verticillioides* infects maize, causing ear rot, yield loss and contamination by fumonisin mycotoxins. The fungus can be transmitted via kernels and cause systemic infection in maize. Maize resistance to the fungus may occur at different developmental stages, from seedling to maturity. Resistance during kernel germination is part of the plant-pathogen interaction and so far this aspect has not been investigated. In the present study, a genome wide association study (GWAS) of resistance to *Fusarium* during the seedling developmental stage was conducted in a maize diversity panel using 226,446 SNP markers. Seedling germination and disease phenotypes were scored on artificially inoculated kernels using the rolled towel assay. GWAS identified 164 SNPs significantly associated with the traits examined. Four SNPs were associated with disease severity score after inoculation, 153 were associated with severity in asymptomatic kernels and 7 with the difference between the severity ratings in inoculated and non-inoculated seeds. A set of genes containing or physically near the significant SNPs were identified as candidates for *Fusarium* resistance at the seedling stage. Functional analysis revealed that many of these genes are directly involved in plant defense against pathogens and stress responses, including transcription factors, chitinase, cytochrome P450, and ubiquitination proteins. In addition, 25 genes were found in high linkage disequilibrium with the associated SNPs identified by GWAS and four of them directly involved in disease resistance. These findings contribute to understanding the complex system of maize-*F. verticillioides* and may improve genomic selection for *Fusarium* resistance at the seedling stage.

*Fusarium verticillioides* (Sacc.) Nirenberg (synonym *Fusarium moniliforme*; teleomorph *Gibberella fujikuroi* MP-A or *Gibberella moniliformis*) (Bömke *et al.* 2008) is a soil and seed-borne fungal species that can be transmitted to and by seeds, respectively (Wilke *et al.* 2007), causes root and stalk infection, and can reach the developing kernels (Bacon and Hinton 1996; Munkvold *et al.* 1997; Munkvold and Desjardins 1997; Munkvold 2003a; Reid *et al.* 2009; Lanubile *et al.* 2010, 2017; Ju *et al.* 2017). As a consequence, *F. verticillioides* incites several diseases including seedling blight, root rot, stalk rot, kernel rot, and ear rot (Munkvold *et al.* 1997; White 1999).

*Fusarium* infection can result in reduced grain yield and poor grain quality. *F. verticillioides* infection is also a serious concern because it produces fumonisins, a family of mycotoxins which have been implicated in several diseases in livestock and humans (Marasas 1996; Presello *et al.* 2008). The best method for controlling *F. verticillioides* infection and fumonisin contamination is through the development and deployment of disease resistant maize genotypes. However, totally immune genotypes are not available and commercial hybrids have less resistance than desired (Lanubile *et al.* 2010, 2017; Zila *et al.* 2013; Ju *et al.* 2017; Maschietto *et al.* 2016, 2017).

Most studies concerning host resistance to *F. verticillioides* have focused on the resistance on *Fusarium* ear rot (FER) in maize. Phenotypic evaluation of FER disease is usually performed in field trials using natural and artificial inoculation techniques (Lanubile *et al.* 2011, 2014a; Zila *et al.* 2013; Ju *et al.* 2017; Maschietto *et al.* 2017). Nevertheless, field evaluation is time consuming, labor intensive, and is effective only when environmental conditions are favorable for disease development. Moreover, to precisely compare levels of quantitative disease resistance among varieties, evaluations must be replicated across different environments in different years (Munkvold 2003a; Robertson *et al.* 2006; Robertson-Hoyt *et al.* 2006; Zila *et al.* 2013; Maschietto *et al.* 2017).

As an alternative to field tests of FER, the rolled towel assay (RTA) is a bioassay used for testing the ability of different pathogens to infect and colonize seedlings. RTA has been previously and successfully applied in both soybean and maize genotypes to evaluate the resistance to *Fusarium* spp. (Ellis *et al.* 2011; Lanubile *et al.* 2015; Bernardi *et al.* 2018). So far, the genetic architecture of resistance to *Fusarium* infection of seedlings (FIS) has not yet been investigated using the RTA.

A number of distinct population structures are currently available for genetic analysis in maize, including biparental recombinant inbred line (RIL) populations obtained by crossing two parental inbreds (Maschietto *et al.* 2017), Multi-parent Advanced Generation Intercrosses (MAGIC) populations (Dell’Acqua *et al.* 2015), the Nested Association Mapping (NAM) population (McMullen *et al.* 2009), and various association mapping panels (Yang *et al.* 2010; Warburton *et al.* 2013; Ju *et al.* 2017). The maize core diversity panel, sometimes referred to as the “Goodman” association panel (Flint-Garcia *et al.* 2005; Zila *et al.* 2013), is a collection of 302 inbred lines capturing much of the maize diversity present in public breeding programs worldwide. This population is composed of current and historically important lines originating from tropical and temperate maize growing areas. The utility of the panel in identifying molecular markers, quantitative trait loci (QTL), and candidate genes underlying complex traits has been previously investigated (Flint-Garcia *et al.* 2005; Cook *et al.* 2012; Zila *et al.* 2013; Zila *et al.* 2014; Olukolu *et al.* 2013, 2016; Samayoa *et al.* 2015).

Genome wide association study (GWAS) is a genomics-based strategy for crop improvement and can be applied to a population of unrelated genotypes, capturing a considerable portion of species variation. Examining the genome-wide associations between single nucleotide polymorphisms (SNPs) and the phenotypes of a desired trait allows the identification of loci for quantitative traits and is especially useful when combined with QTL mapping (Brachi *et al.* 2011). GWAS was performed both in the maize core diversity panel (Zila *et al.* 2013) and NCRPIS collection of inbred lines which includes the maize core diversity panel (Zila *et al.* 2014) in order to detect SNPs associated with increased resistance to FER, and revealed 10 SNPs with significant effect on several chromosomes (Zila *et al.* 2013, 2014). However, the effects of these SNPs were relatively small, suggesting that the introgression of few specific resistance loci may not have a large overall impact on resistance levels within temperate breeding population complex.

The first objective of this study was to evaluate phenotypically the maize “Goodman” association population for resistance to FIS using the RTA bioassay and considering the effects of the natural and artificial infection (SEV_C and SEV_I, respectively), and the differences between them (Fv_ADD). SEV_C may contribute to seed infection because *F. verticillioides* can be transmitted from seed to seedling (Munkvold 2003b). In fact, maize fields are almost naturally and universally infected by *F. verticillioides*: the fungus is commonly isolated from asymptomatically, apparently sound kernels and it is difficult to find grain lots in which the pathogen is not associated with at least a small percentage of kernels. In order to obtain a better estimation of the effect of the artificial infection, Fv_ADD was considered by subtracting the value for natural infection (SEV_C) from that of artificial infection (SEV_I). Indeed, a high natural infection severity could indicate the presence of the fungus inside the seed of some lines, masking the effect of the artificial inoculation. As second objective of the work, the three traits (SEV_C, SEV_I and Fv_ADD) were considered to perform GWAS and linkage disequilibrium (LD) analyses to identify SNPs associated with resistance to *F. verticillioides* infection in seedlings.

## Materials and Methods

### Maize germplasm

The “Goodman” maize association population (Flint-Garcia *et al.* 2005) was evaluated for *F. verticillioides* resistance. Seeds were retrieved from USDA-ARS-NCRPIS (Iowa State University, Regional Plant Introduction Station, Ames, Iowa, United States, 50011-1170). Due to seed availability, only 265 out of 302 inbred lines of the population were screened (Table S1), and after excluding lines with <50% germination percentage, 230 lines were used for GWAS.

### Experimental procedure for rolled towel assay

For each inbred line, seeds with similar size and shape and without visible damage were selected for the experiment. To reduce as much as possible the presence of contaminating fungi, seeds were surface-sterilized by shaking them in 50-ml tubes at room temperature with 70% ethanol for 5 min, followed by sterile distilled water for 1 min, then with commercial bleach solution for 10 min, and finally rinsed three times with sterile distilled water for 5 min each time. Two towels of germination paper (Anchor Paper, Saint Paul, MN) were moistened with sterilized distilled water. For each inbred, 10 seeds were placed on the germination paper about 10 cm from the top of the towel with the embryo side facing out. Kernels were inoculated on the embryo side near the pedicel with 100 μl of a 1x10^6^ ml^-1^ spore suspension of *F. verticillioides* ITEM10027 (MPVP 294). The strain was isolated from maize in South Tuscany, Italy, by the Department of Sustainable Crop Production, Piacenza, Italy and deposited in their fungal collection and also in the Institute of Science and Food Production, National Research Council of Bari, Italy (http://server.ispa.cnr.it/ITEM/Collection). Another moistened towel was placed over the inoculated seeds, the towels were rolled up and placed vertically in a 25-L bucket covered with a black plastic bag and incubated for 7 days at 25° in the dark. For each inbred, a control RTA was prepared as previously described, replacing the inoculation step with *F. verticillioides* by 100 μl of sterilized distilled water. Inoculated and non-inoculated towels were incubated in common buckets, but kept in separate, open plastic bags to avoid cross-contamination.

### Disease severity evaluation after inoculation

Seedlings were rated using a five-point severity scale as previously described in Bernardi *et al.* (2018). On this scale, 1 = healthy, germinated seedlings with no visible signs of colonization; 2 = germinated seedlings and initial colonization of the kernel near the pedicel; no symptoms on primary and side roots, hypocotyl and first leaf of the seedling; 3 = germinated seedlings with widespread colonization of the kernel and browning of the coleoptile; no symptoms on primary and side roots of the seedling; 4 = germinated seedlings with reduced development, complete colonization of the kernel, and lesions and abundant mold on the shoot; and 5 = no germination due to complete rotting of the kernel (Figure S1**)**. The percent of germinated seeds was recorded for each towel. Thirty-five inbred lines with germination percent below 50% in the control condition were removed from further analysis, since their phenotypes could be conditioned by infections from other pathogens during seed development. SEV_I and SEV_C were recorded for each line as well as the difference between SEV_I and SEV_C (Fv_ADD) was calculated for each line. For each trait, means and standard deviations were calculated. The ANOVA analysis was performed using R software (R Core Team 2016). To estimate the reliability of the assay, a second replication of the experiment was performed for 140 lines with sufficient seeds and the ANOVA analysis was performed to compare the replicates (R Core Team 2016).

### Genotypic data and association analysis

Genotyping by sequencing (GBS) approaches have been used to genotype the complete maize association panel (Elshire *et al.* 2011; Glaubitz *et al.* 2014). The original data set (ZeaGBSv2.7) comprises 955,690 SNP markers partially imputed with AGPv3 coordinates. Monomorphic and multiallelic SNPs and INDELs were removed. Additional imputation was performed using the software Beagle 4.1 (Browning and Browning 2016). After setting heterozygotes as missing data and removing those SNPs with > 20% missing and minor allele frequency (MAF) <5% after the final imputation, a set of 226,446 SNPs was used to perform association mapping.

Linkage disequilibrium-based pruning was performed using the software Plink v1.07 (Purcell *et al.* 2007). Markers with genotypic correlations greater than *r* = 0.5 were pruned. This step yielded a subset of ∼100 kb SNPs used to compute the realized additive relationship matrix (**K** matrix) for the set of 230 lines by using TASSEL v5.2.25 (Bradbury *et al.* 2007).

Genome Wide Association Analysis (GWAS) was performed in TASSEL (version 5.2.25). The mixed linear model (MLM) fitted was:

y=Xβ+Zu+e

where **y** is the vector of phenotypes, **β** is a vector of the overall mean and the fixed effect estimate of an individual SNP, ***u*** is a vector of random line additive genetic background effects, **X** and **Z** represent incidence matrices, and ***e*** is a vector of random residuals. Variance of random line effects was modeled as Var (u) = **K**$\sigma_{a}^{2}$, where $\sigma_{a}^{2}$ is the estimated additive polygenic variance. The optimum compression level option (compressed MLM) was used (Zhang *et al.* 2010).

To identify robust SNP associations, a resampling GWAS procedure was performed (Valdar *et al.* 2009). In each of 100 data resamples, a random sample of 80% of inbred lines was selected from the diversity population and GWAS was performed on the subset of lines. Only SNP markers determined as significant at *P* < 1×10^−4^ within at least 30% of data subsamples, *i.e.*, a resample model inclusion probability (RMIP) threshold of 0.30, were considered as significant (Samayoa *et al.* 2015). Data manipulations and visualizations were performed using R software (R Core Team 2016).

### Candidate gene discovery, SNP variant effects, and linkage disequilibrium analysis

Candidate genes containing, or adjacent to associated SNPs were identified using the Maize GDB genome browser. The B73 RefGen_V3 was the reference genome used to localize SNPs and genes. Transposable elements and low-confidence genes were not considered. Where possible, the predicted function of the gene was recorded. All proteins encoded by the genes were submitted to NCBI-protein BLAST (https://blast.ncbi.nlm.nih.gov), restricting the search to *Zea mays* (taxid:4577) protein function; conserved domains identified in this way were recorded.

SNP positions inside genes were identified and the possible effect of each trait-associated SNP on protein sequence was investigated. To detect introns and translated regions, genic and protein sequences were submitted to GeneWise online tool (http://www.ebi.ac.uk). Coding sequence translation was performed using the translate tool available from ExPASy (http://web.expasy.org/translate), and amino-acid variations caused by the SNPs were detected by alignment with the corresponding reference protein available from Maize GBD (http://multalin.toulouse.inra.fr).

Linkage disequilibrium (LD) measures (*r*^2^) were estimated between each SNP significantly associated with resistance traits and all other SNPs within a surrounding window of 60 adjacent SNPs using TASSEL (Samayoa *et al.* 2015). For clusters of tightly-linked significant SNPs, LD analysis was computed for one of them. Positions were considered in high LD with significant SNPs if *r*^2^ > 0.5 and the distance between the position and the SNP resulting from GWAS was greater than 2 Kb; the gene containing or closed to these new positions was recorded.

### Data availability

All supporting data are included as supplemental files and are available at Figshare: https://doi.org/10.6084/m9.figshare.c.4162766. Table S1. (DOCX file) List of germplasm and phenotypic values. Figure S1. (PDF file) Disease severity scale. Table S2. (DOCX file) Table of SNPs resulting significantly associated to the traits from GWAS analysis. Table S3. (DOCX file) Table of genes associated to significant SNPs from GWAS analysis. Table S4. (DOCX file) Table of LD analysis. Figure S2. (PDF file) Localization of genes and QTL for resistance on Chromosomes 1, 2 and 3. Figure S3. (PDF file) Localization of genes and QTL for resistance on Chromosomes 4, 5 and 6. Figure S4. (PDF file) Localization of genes and QTL for resistance on Chromosomes 7, 8, 9 and 10. 

## Results and Discussion

### Infection severity

**Figure 1:**
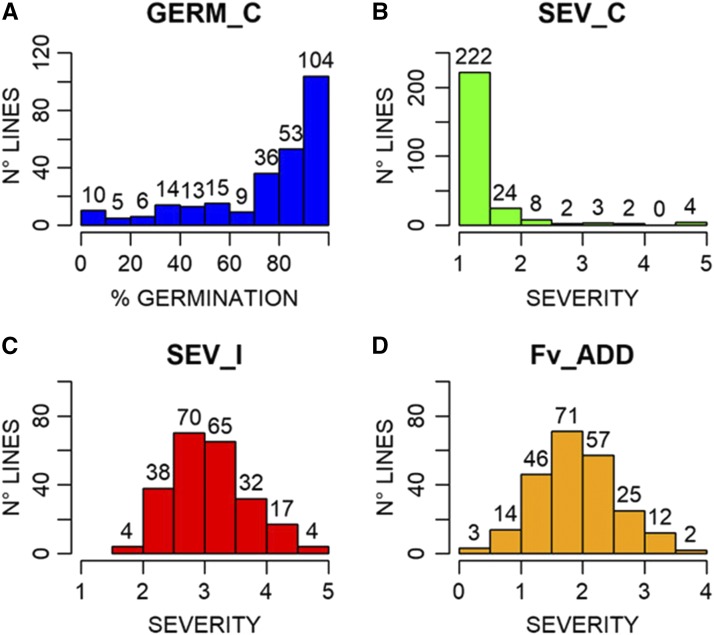
Germination (in percentage, GERM_C; A) and disease severity rate (B) in the control rolled towel assays of kernels of 267 maize lines. Disease severity rate after *Fusarium verticillioides* inoculation (SEV_I; C). Disease severity rate as difference between *F. verticillioides* inoculated and control kernels (Fv_ADD; D). The number of lines for the different classes of germination and severity is indicated above each histogram.

Different types of response to FIS were found in the association maize panel (Figure 1). The phenotypic values for SEV_C, SEV_I and Fv_ADD, calculated as mean of ten kernels per each line and their standard deviations, are reported in Table S1a. ANOVA showed high significant differences among lines and treatments (Table S1b). The ANOVA performed for the partial replication of RTAs in a subset of 140 inbred lines showed significant differences among lines (p-value <2*10^−16^), but not between replications (p-value 0.172; Table S1c), demonstrating that the assay is repeatable. Indeed, the correlation between replicates was high (r = 0.91).

Germination scores revealed that 104 lines had more than 95% germination and 157 had more than 80% germination (Figure 1A). Lines with less than 50% germination in the control experiment were eliminated, leaving 230 lines for further analysis. Since corn kernels do not have a smooth surface, the sterilization protocol is necessary to remove, as much as possible, contaminant fungi already present on the surface of the kernel asperities. Despite the sterilization protocol, *F. verticillioides* was observed in some non-inoculated kernels, indicating natural symptomless infection inside the kernels (Munkvold and Desjardins 1997). More than 90% of the non-inoculated lines had a mean severity score under 2, but 11 inbreds showed higher severity rates (Figure 1B and Table S1).

*F. verticillioides* inoculation resulted in 135 inbreds with mean severity score between 2.5 and 3.5 (58% of the remaining lines). Extreme values, less than 2 (low infection) or greater than 4.5 (high infection), were observed for 4 inbreds in each tail of the distribution (Figure 1C). The inoculation was effective, as no lines had mean severity less than 1.5 and no completely healthy seedlings were found in the inoculated towels. The distribution of Fv_ADD (Figure 1D), was similar to that observed for SEV_I: 128 lines had a change in severity score between 1.5 and 2.5 (55.6% of the remaining lines) due to inoculation.

**Figure 2:**
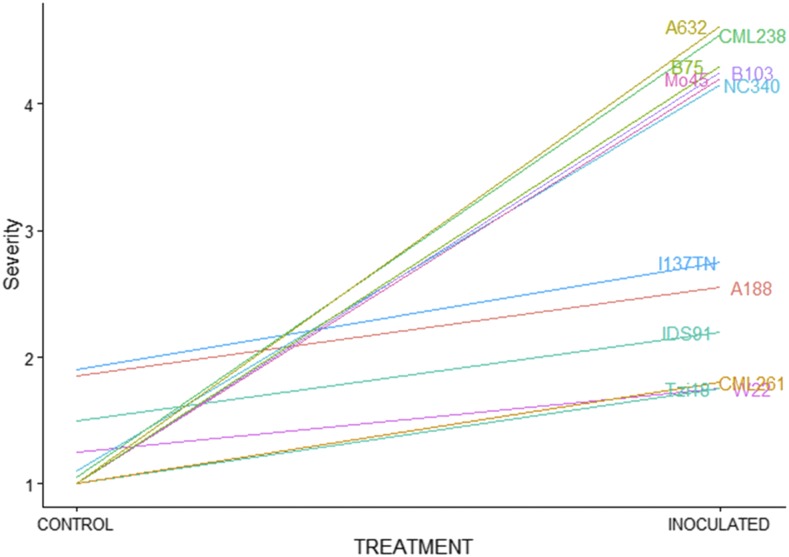
Effect of inoculation with *Fusarium verticillioides* on disease severity rate, compared to control, in kernels of selected maize lines.

Three lines had a severity increases of less than 0.5: CML254 with a SEV_C of 2.35 had no increase; P39 increased from SEV_C of 2.15 to SEV_I of 2.45, and W22 increased from SEV_C of 1.25 to SEV_I of 1.75 (Figure 2). The greatest increases in disease severity due to inoculation were observed for I29 with SEV_C of 1 and SEV_I of 4.8, followed by A632 with SEV_C of 1 and SEV_I of 4.61 (Figure 2). Some lines with the lowest response to inoculation were also found to be contaminated in the control assays.

### Association analysis and SNP discovery

GWAS was performed on 230 inbred lines and revealed a total of 164 SNPs, distributed across all chromosomes, significantly associated in at least 30% of data samples with the three phenotypic traits examined (SEV_C, SEV_I and Fv_ADD). One hundred and eleven SNPs were located in 75 genes, while the remaining 53 SNPs were intergenic, but within 61 kb from known genes. A total of 101 genes, containing or close to SNPs significantly associated with at least one of the measured traits were identified. Eleven SNPs occurred inside two different genes each, as two genes were annotated in the same region on complementary strands. A summary of SNPs and gene numbers, associated with SEV_C, SEV_I or Fv_ADD, is reported in Table 1 along with the corresponding chromosome and the number of intragenic and intergenic associated SNPs. The entire list of the individual SNPs associated to the traits is reported in Table S2.

**Table 1:** Chromosome location, number of genes and SNPs and number of SNPs localized inside (IN) or outside (OUT) genes significantly associated with the traits SEV_C, SEV_I, Fv_ADD

TRAIT	CHR	GENES	SNPs	IN	OUT
**SEV_C**	1	10	13	6	7
**SEV_C**	2	13	31	23	8
**SEV_C**	3	18	21	17	4
**SEV_C**	4	9	9	7	2
**SEV_C**	5	8	20	5	15
**SEV_C**	6	8	10	5	5
**SEV_C**	7	9	13	9	4
**SEV_C**	8	11	16	11	5
**SEV_C**	9	6	13	12	1
**SEV_C**	10	3	7	7	0
**SEV_I**	3	1	1	0	1
**SEV_I**	4	1	2	2	0
**SEV_I**	7	1	1	1	0
**Fv_ADD**	1	1	5	5	0
**Fv_ADD**	2	1	1	0	1
**Fv_ADD**	6	1	1	1	0

**Figure 3:**
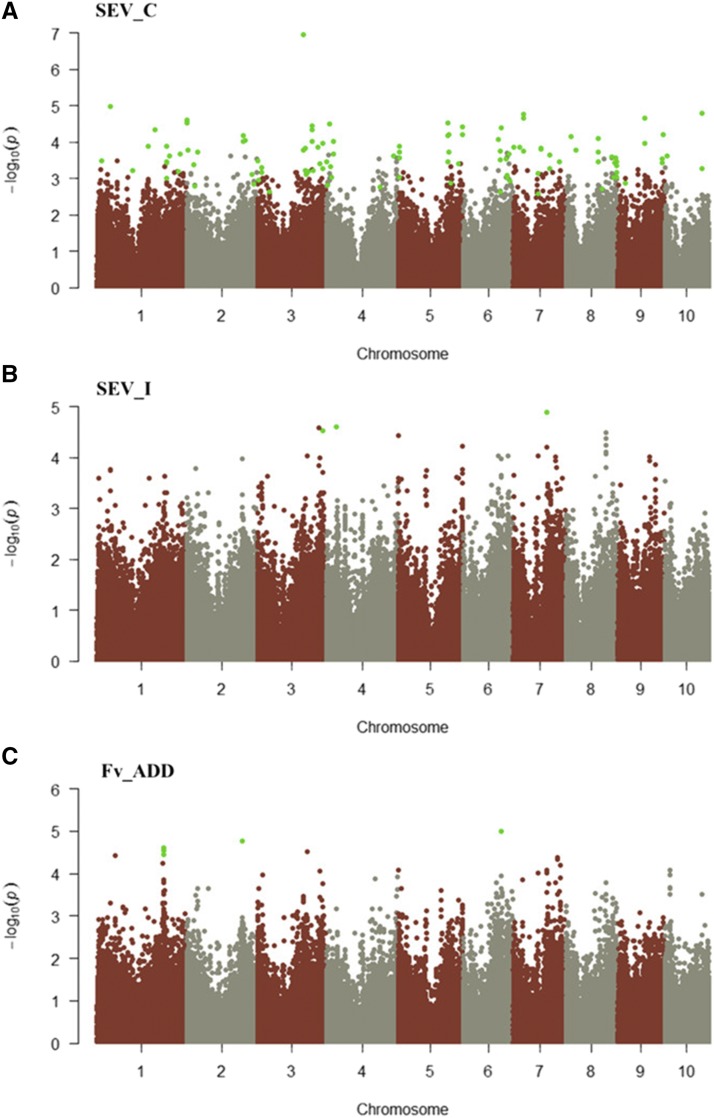
Manhattan plot for the trait SEV_C (A), SEV_I (B) and Fv_ADD (C). SNPs significantly associated to the trait are indicated by green dots.

For the trait SEV_C, 153 SNPs were found significantly associated and localized on all the chromosomes (Figure 3A). The majority of these SNPs (31) were on chromosome 2, while chromosome 10 had the lowest number (7). Among these SNPs, 102 localized inside genes, while 51 were located outside (Table S2).

**Figure 4:**
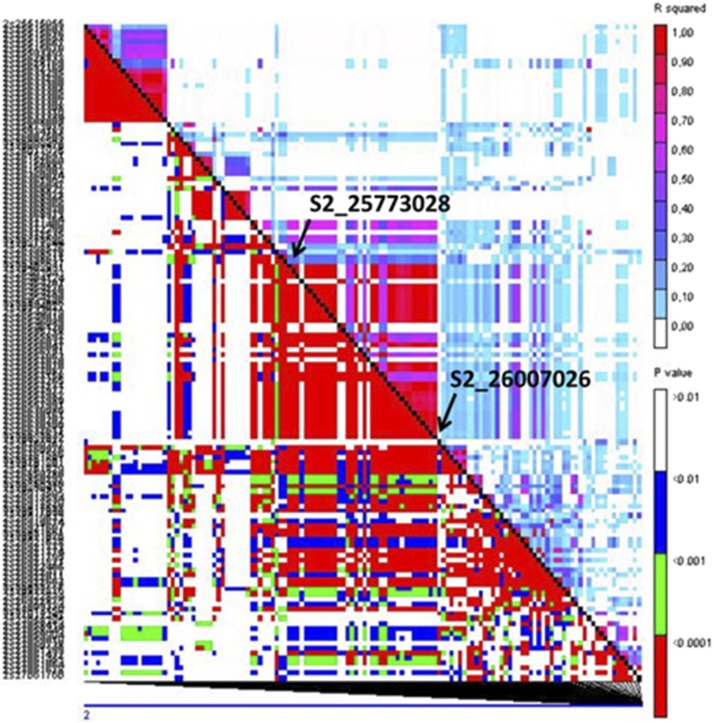
LD heatmap showing LD measure (*r*^2^) calculated for each pairwise combination of SNPs surrounding each SNP significantly associated with resistance traits. Colors indicate the magnitude of each pairwise *r*^2^ measure (*r*^2^ = 1 red to *r*^2^ = 0 white). The LD-block on chromosome 2 is reported. LD was computed in a window of 134 markers: 50 upstream and 50 downstream of the flanking SNPs evidenced by GWAS. Arrows indicate the position of interest.

For SEV_I, 4 SNPs were found significantly associated and localized on chromosomes 3, 4 and 7 (Figure 3B). Two SNPs on chromosome 4 and one SNP on chromosome 7 were found inside a gene, whereas the associated SNP on chromosome 3 was found outside the gene. For the trait Fv_ADD, 7 SNPs were found on chromosomes 1, 2 and 6 (Figure 3C). Five and one SNPs were located inside a single gene on chromosomes 1 and 6, respectively; the SNP on chromosome 2 localized to an intergenic region 128 bp in proximity of the gene (Figure 4).

### Genes associated with the trait SEV_C

SNPs associated with SEV_C were found in 95 genes. Among these, 30 are reported to have a function that can be connected to disease resistance mechanisms (Table S3). Four of these candidate genes are discussed as follows.

Five significantly associated SNPs are in the coding sequence of the gene GRMZM2G005633 on chromosome 10, which encodes a chitinase B1 protein (from 22 to 26 in Tables S2 and S3). Four of the SNPs are predicted to change amino acids in the protein encoded by transcript 2 (T2) of the gene: N159K (Asparagine-Lysine), N159T (Asparagine-Threonine), A160T (Alanine-Threonine) and Y161H (Tyrosine-Histidine). These allelic variations are in the catalytic residues of the Glycoside hydrolase family 19 (chitinase) domain and they could have a significant impact on the protein function. Among the sequences currently available online, amino acid variability is reported for positions 159 and 160, while the Tyrosine 161 appears to be highly conserved (Marchler-Bauer *et al.* 2015). Classes I, III and IV of the glycoside hydrolase family 19 are mainly described in plants. These enzymes are responsible for the hydrolysis of the beta-1,4-N-acetyl-D-glucosamine linkages in chitin polymers. Chitin is a common component of fungal cell walls and the exoskeleton of arthropods. Chitinase preparations are able to inhibit the growth of fungi since they cause the lysis of hyphal tips and are able to accumulate around hyphal material *in planta* (Collinge *et al.* 1993). Chitinases are induced in plants exposed to various stresses, indicating that these enzymes are produced as part of a general stress response; it is also possible that they are an active component of the plant defense system (Collinge *et al.* 1993). The combination, in the same plant elite line, of chitinase genes with others encoding antifungal products has been proposed as a strategy to reduce the need to spray with antifungal chemicals (Collinge *et al.* 1993). Germinating maize embryos produce two acidic chitinase isozymes as response to *F. moniliforme* infection and a chitinase from inbred line Tex6 inhibits the growth of *Aspergillus flavus* (Hawkins *et al.* 2015). This chitinase is highly similar to the homologous chitinases A (encoded by GRMZM2G051943) and B (encoded by GRMZM2G005633). Commercial hybrids of maize produce two isoforms of the chitinase B that appear to be modified by fungi reducing the overall chitinase activity (Hawkins *et al.* 2015).

On chromosome 2, the gene GRMZM2G149996 encodes an aspartic protease localized to guard cells. The protein contains two different conserved domains: a pepsin-like aspartate protease and a TAXi_C (Xylanase inhibitor C-terminal). The N- and C-ends of the proteins in this family are necessary to create the catalytic pocket required to split xylanases. Phytopathogens produce xylanases that destroy the hemicellulose in the cell walls, whereas the ability of host plants to proteolyze xylanases is vital for plant-survival. Two significant SNPs (28-29 in Tables S2 and S3) were found inside this gene; one is responsible for the change G225S in the catalytic residue of the enzyme (Marchler-Bauer *et al.* 2015).

A significant SNP on chromosome 3 is in the gene GRMZM2G132509 coding for a xylanase inhibitor TAXI-IV precursor (Tables S2 and S3**)**. TAXI proteins occur in several species where they are induced by pathogens and wounding. Moreover, TAXI proteins are active against xylanases produced by *F. graminearum* (Fierens *et al.* 2007). The SNP localized inside this gene is not predicted to cause an amino acid variation.

Another significant SNP is in the Untranslated Terminal Region (UTR) of GRMZM2G160027 on chromosome 5 (Tables S2 and S3**)**. This gene encodes for a glycine rich protein (GRP) A3. These proteins are structural component of the cell walls of many higher plants (Ringli *et al.* 2001). The expression of these genes is regulated by several biotic and abiotic factors. The involvement of GRP in plant defense against pathogens is reported for tobacco, *Arabidopsis* and *Capsella bursa-pastoris* (Mangeon *et al.* 2010). A glycine rich cell wall structural protein is encoded also by GRMZM2G149446 that was highlighted by SNPs n° 13-16 on chromosome 1 (Tables S2 and S3).

The results of this study confirm that several genes may have a role in response to infection with endemic strains of *F. verticillioides*. The infected kernels are frequently symptomless, but they contribute to the contamination of seedlings during development anyway (Yates and Jaworski 2000). Even though it cannot be excluded that other fungi may be associated with *F. verticillioides*, the findings about SEV_C from this study suggest that the symptomless infection should be taken in account and targeted to prevent seedling infection.

### Genes associated with the trait SEV_I

SNPs in three genes were associated with SEV_I: GRMZM2G087628, encoding the plastid S1-binding domain protein; GRMZM2G029153, encoding for a protein with domains common to sugar carriers and transporters (Marchler-Bauer *et al.* 2015); and GRMZM2G087628, which encodes a putative cytochrome P450 (Tables S2 and S3**)**. In plants, cytochrome P450s are involved in hormone metabolism, the oxygenation of fatty acids for the synthesis of cutins, lignification, and the synthesis of secondary metabolites (Werck-Reichhart and Feyereisen 2000). The deposition of cutins and lignification are important mechanisms in plant defense against pathogens. Recently, Yang *et al.* (2017) identified a gene encoding caffeoyl-CoA *O*-methyltransferase, involved in lignification, as able to confer quantitative resistance to multiple foliar maize pathogens. Moreover, cytochrome P450s are deeply involved in the degradation of environmental toxins and mutagens. The observed two polymorphisms were in the coding sequence of the gene and one is responsible for an amino acid variation (V231I) occurring inside the conserved domain of the protein.

### Genes associated with the trait Fv_ADD

SNPs in three genes were significantly associated with variation for Fv_ADD: GRMZM2G078088, GRMZM2G113257 and GRMZM2G163054 (Tables S2 and S3). The latter is on chromosome 6 and encodes for a putative WRKY125 transcription factor. WRKY proteins have a regulatory function in several plant metabolisms and in transcriptional reprogramming as a response against pathogens and abiotic stresses (Rushton *et al.* 2010). Targets of WRKY proteins are W-boxes, WRKY genes, and defense related genes of the pathogenesis related (PR) type. WRKY proteins are also involved in gibberellic and jasmonic acid response (Eulgem *et al.* 2000). In developing maize kernels after *F. verticillioides* and *A. flavus* infection, the WRKY125 transcription factor was observed to be highly expressed (Lanubile *et al.* 2014b; Shu *et al.* 2015). The SNP is located inside a coding sequence but is not responsible for an amino acid change. GRMZM2G113257 encodes for the transcription factor bHLH 169, whereas GRMZM2G078088 encodes for a hypothetical protein.

### Linkage disequilibrium analysis

Linkage disequilibrium analysis was performed to identify correlations between markers due to cosegregation or population structure. In diverse maize, LD generally decays to very low levels on average between 5 and 10 Kb, with some variation observed among chromosomes (Yan *et al.* 2009). LD can be more extensive around genes that have been targets of selection sweeps (Remington *et al.* 2001). In this work, 35 SNPs or groups of SNPs had linkage disequilibrium *r*^2^ ≥0.5 with significantly associated markers. These additional markers were in or adjacent to 40 genes, 14 of which were part of the gene set directly identified from GWAS (Table S4). Seven genes identified by LD analysis have an annotation which can be connected with disease resistance mechanisms (Table S4).

### Localization of genes within QTL for resistance

To investigate the possible overlap between genes for the resistance to FIS resulting from GWAS and previously reported QTL for FER, the genes containing or adjacent to significant SNPs were located on chromosomes according to physical positions, and QTL important for FER and fumonisin accumulation resistance (Lanubile *et al.* 2014a 2017; Maschietto *et al.* 2017) were located according to bin positions, as shown in Figures S2, S3, and S4.

In several cases it was possible to find overlaps between genes and known QTL. For SEV_I, the gene GRMZM2G087628 was found in QTL reported both for FER and fumonisin accumulation resistance on chromosome 3; the gene GRMZM2G037781 on chromosome 4 was located in a QTL for fumonisin accumulation resistance, while on chromosome 7 GRMZM2G029153 was found in a region where QTL for both FER and fumonisin accumulation resistance overlapped.

For SEV_C, among the four identified genes involved in the resistance mechanisms, all overlapped with QTL for fumonisin accumulation on chromosomes 2, 3, 5 and 10.

For Fv_ADD only the gene GRMZM2G163054 overlapped a QTL for fumonisin accumulation on chromosome 6.

The “Goodman” association population was previously tested in field experiments for resistance to FER elicited by *F. verticillioides* (Zila *et al.* 2013). Three associated SNPs in three different genes were reported. Six genes were found based on GWAS in a much larger panel containing the entire public maize inbred line collection of the USA screened for FER (Zila *et al.* 2014). These genes were all different from those found in this study and were not close to any of them. This may be a consequence of the different experimental approaches (field *vs.* laboratory conditions) and measurement of different disease phenomena (FER *vs.* FIS resistance). Moreover, resistance to ear rot in the field is also expected to be influenced by different characteristics, such as ear architecture traits, including husk tightness, ear coverage, and differences in flowering and maturing times (Flint-Garcia *et al.* 2005; Sweets and Wright 2008; Santiago *et al.* 2015).

Six of the genes associated with SEV_C in this study were previously found to be up-regulated in maize ears after infection with *F. verticillioides* (Lanubile *et al.* 2014b). Genes GRMZM2G071484 and GRMZM2G125762, encoding for an ubiquitin-protein ligase and a protein kinase, respectively, were found as common differentially expressed genes after *F. verticillioides* inoculation in CO441 and CO354 maize inbreds. GRMZM2G036564 and GRMZM2G425629, coding for a transmembrane protein 20 characterized by the PGG domain and late embryogenesis abundant protein, respectively, were observed as specific of the resistant line CO441. Whereas AC233899.1_FG004 encoding for a leucine zipper transcription factor involved in signal transduction and GRMZM2G092817 encoding a 4,5-DOPA dioxygenase extradiol were specific of the susceptible CO354 line.

In a recent work, Ju *et al.* (2017) reported 8 QTL associated with *Fusarium* seed rot (FSR) in a RIL population, which was evaluated with a different phenotyping method. Nine genes associated with SEV_C identified in the present work were located inside three of these QTL.

On chromosome 2, GRMZM2G062471, AC218093.3_FG005, GRMZM2G376731 and GRMZM2G357972 were located in the QTL qFR2-1 Ju *et al.* (2017). On chromosome 3, inside the QTL qFR3, genes GRMZM2G027375, GRMZM2G098042 and GRMZM2G042364 were found, while the genes GRMZM2G004440 and GRMZM2G140822 were located inside qFR8 QTL on chromosome 8. On the other hand, no correspondence was found with the genes reported for FSR and those found in the present work. A possible explanation is the fact that in RTA, seeds were widely able to germinate also after the inoculation, while in Ju *et al.* (2017) work, seeds did not germinate after *F. verticillioides* inoculation. In the case of a non-germinating seed the fungus can grow as a saprophyte, conversely, in the RTA bioassay the living seedlings were able to counteract fungal attack.

### Conclusions

A RTA bioassay was applied in this study for the first time to screen the “Goodman” maize association population for FIS resistance. GWAS analysis revealed 164 SNPs associated with 3 phenotypic traits (SEV_C, SEV_I, Fv_ADD) that are the direct measure of fungal growth on the kernel. Globally, 101 genes were found associated to the SNPs and many of these genes have a role in disease response. LD analysis among markers revealed 23 additional genes, 3 of those are directly involved in mechanism of pathogen defense. Co-localization between genes described in GWAS analysis and QTL reported for FER or fumonisin accumulation resistance revealed that the majority of the genes associated with the SNPs, localized inside QTL for FER and/or fumonisin accumulation resistance traits. The high number of markers identified and the small effect of each marker on disease severity is consistent with the quantitative nature of the *F. verticillioides*-maize pathosystem. Nonetheless, these new markers may be useful to improve genomic selection for identifying new maize genotypes adequate to counteract *F. verticillioides* infection at the seedling stage.
